# Global marine fish trade networks track international pathways of nutrients and contaminants

**DOI:** 10.1016/j.eehl.2026.100218

**Published:** 2026-01-29

**Authors:** Yiou Zhu, Quang Tri Ho, James P.W. Robinson, Marian Kjellevold, Ruirong Chang, Edvin Fuglebakk, Jianmin Ma, Shijie Song, Lisbeth Dahl, Ole Jakob Nøstbakken, Maria W. Markhus, Bente M. Nilsen, Tanja Kögel, Anne-Katrine Lundebye, Atabak M. Azad, Abimbola Uzomah, Jeppe Kolding, Vidar S. Lien, Martin Wiech, Yanxu Zhang, Amund Maage, Livar Frøyland, Michael S. Bank

**Affiliations:** a Institute of Marine Research, Bergen 5817, Norway; b Lancaster Environment Centre, Lancaster University, Lancaster, LA1 4YQ, United Kingdom; c Joint International Research Laboratory of Atmospheric and Earth System Sciences, Nanjing University, Nanjing 210093, China; d College of Urban and Environmental Sciences, Peking University, Beijing 100871, China; e College of Earth and Environmental Sciences, Lanzhou University, Lanzhou 730000, China; f Federal University of Technology, PMB 1526, Owerri, Nigeria; g University of Bergen, Bergen 5020, Norway; h Department of Earth and Environmental Sciences, Tulane University, New Orleans, LA 70118, US; i University of Massachusetts Amherst, Amherst, MA 01003, US

**Keywords:** Global trade, Food security, Nutrients, Mercury, Dioxin, Human health

## Abstract

Marine fish trade globalizes nutrients and contaminants. Using trade data, human demographic information, and nutrient and contaminant exposure data, the estimated direct consumption of traded fish from Northeast Atlantic Ocean (NEAO) catches varied among 155 importer countries/regions. The associated trade pathways globalised high amounts of important nutrients including iodine, selenium, and eicosapentaenoic acid and docosahexaenoic acid (EPA + DHA) and contributed greatly to annual domestic EPA + DHA requirements for small-population importers (e.g., Lithuania: 62.8%) but not for high-population importers (e.g., Chinese mainland). Traded amounts of mercury, dioxin, and dioxin-like polychlorinated biphenyls (dl-PCBs) from the NEAO fish were low, and associated pathway contributions to total domestic mercury exposures were <4%. Changes in fish body size affected nutrient and contaminant fillet concentrations and subsequently trade dynamics of nutrients and contaminants. Our study provides valuable insights regarding seafood globalization and marine fish trade that can be used to support adaptive management strategies for contaminants and nutrition-sensitive policies.

## Highlights

• International wild fisheries trade globalized nutrients and contaminants.
• The Northeast Atlantic fishery generally had high nutrients and low contaminants.
• Fish imports improved nutritional security for some small-population countries/regions.
• Fish body size fluctuations modulated global trade dynamics of nutrients and contaminants.

## Graphical abstract

**Figure:**
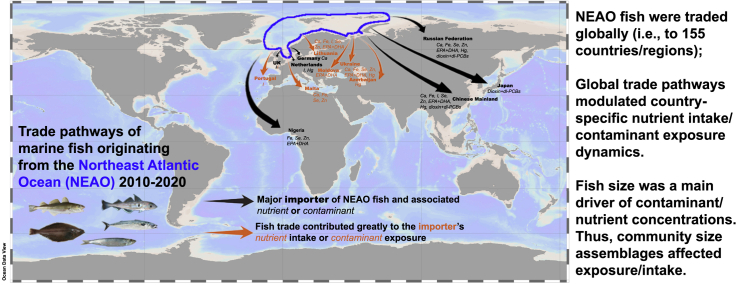

## 1 Introduction

Most countries/regions supply important nutrients from domestic food production [1] but no more than 1% of food production is aquatic [2] with an even lower percentage via international trade. Domestic food production varies across countries/regions [3], but international food trade has been growing [4], which has resulted in increasing global aquatic food consumption between 1976 and 2019 [5], and with demand for foods from the ocean predicted to double by 2050 [6]. Marine fish are a major component of aquatic foods and an important source of essential nutrients for human consumers [7,8] and are foundational in improving health and maintaining daily functions [9,10]. These locally produced nutrients are distributed globally via international trade [4,11] and play an important role in combatting micronutrient deficiencies or “hidden hunger” [[12], [13], [14], [15], [16]]. Although there has been a growing interest in food nutrients (as opposed to weight-based monetary value [17]) and fisheries (e.g., the concept of maximum nutrient yields [18]), relevant national and international public policies largely do not consider aquatic foods and their nutrition potential in a food security context [19,20]. For example, only the Philippines (2012) and Cambodia (2017) include national dietary guidelines identifying fish as an important source of calcium (FAO Dietary Guidelines). Although there are regulations on nutrition claims for food (e.g., EU) which can be applied to fish in a global trade context, no import regulations currently prioritise essential nutrient intakes.

Health impacts from marine fish are regularly debated due to the presence of contaminants and biological hazards [9,10,[21], [22], [23]] and are often more visible in national and international policies compared to nutrients. Contaminants are also distributed globally via international fish trade, e.g., polybrominated diphenyl ethers (PBDEs) [24], polychlorinated biphenyls (PCBs) [25], and mercury [[26], [27], [28]], but how seafood globalization may impact human health remains poorly understood. Thus, to realise the potential of the global fish trade in support of sustainable and nutrition-sensitive aquatic food systems and relevant public policies requires an understanding of global trade pathway dynamics and associated health risks and benefits.

International marine fish trade and tracing of nutrients and contaminants can be analysed using global trade flow data (e.g., UN Comtrade) of marine fish species and products [4] and their chemical composition [11,25,26]. However, the consumption of imported fish and their chemical contents vary among countries/regions [25], resulting in uncertainty in measurements of nutrient intakes and contaminant exposures. Current food composition data often relies on fixed concentrations for species/products, thus limiting accurate estimation of nutrient yields and hindering effective management strategies to address hidden hunger. Therefore, evaluating species-specific variation in nutrient and contaminant concentrations of captured marine fish among important fisheries regions is essential for global fish trade.

An important driver of nutrient and contaminant variation is marine fish body size, with small-sized species [8,29] and small individuals of the same species [30] often being denser in essential nutrients. Additionally, small individuals typically have lower concentrations of many contaminants [[30], [31], [32], [33]]. Body size structure of fish assemblages is often used as an indicator of fishing pressure and is a suitable proxy for ecological sustainability in marine fisheries [34,35]. Globally, fish assemblages are shifting towards smaller size-dominated population structures, often with increased abundance and stable population biomasses due to human exploitation [36]. However, the implications of fish body size on the dynamics of nutrient yields and contaminant exposures have not been assessed in the context of seafood globalization.

Here, we analysed and mapped the global fate of six important nutrients and two critical contaminants from five commonly traded fish species from the Northeast Atlantic Ocean (NEAO) by integrating model-predicted concentrations into trade data. Subsequently, we analysed food balance data, human demographic information, average nutrient requirements, and exposure data to estimate the potential health risks and benefits through direct human consumption. Finally, we combined size-based predicted element concentrations with size structure data to investigate whether size-specific fish catch dynamics influenced the total element yields.

We hypothesized that the traded nutrients and contaminants would be species-dependent and governed by mass compositions for each trade pathways, and that the associated risks and benefits were also modulated by fish imports and human demography. We also predicted that body size structure of fish populations would be an important driver, affecting nutrient yields and contaminant exposures with potential additive effects on consumer health.

## 2 Methods

### 2.1 Model-predicted nutrient and contaminant concentrations

The concentrations of five essential elements (*ee* [Table S1 for abbreviations]; calcium, iron, iodine, selenium, and zinc; in mg/kg wet weight [ww]) and one hazardous element (mercury; in mg/kg ww) in five commonly traded marine fish species (*s*) from the NEAO were predicted using previously reported Bayesian predictive models [30]. The fish species analysed were Atlantic cod (*Gadus morhua*), haddock (*Melanogrammus aeglefinus*), Greenland halibut (*Reinhardtius hippoglossoides*), Atlantic herring (*Clupea harengus*), and Atlantic mackerel (*Scomber scombrus*). The model for each element concentration driven by fish total length, fat content, sea temperature, sea salinity, and associated ocean basin was developed by analysing a large dataset (model parameter details in Table S2). Predictions were based on specific ocean basins or regional seas identified for each species and ocean basin-specific total length ranges, while other parameters (fat content, sea temperature, and salinity) were assumed to be the species-mean from all samples from the respective ocean basin (Table S3a).

To explore human element intake from fish consumption, the wet weight concentrations in 100 g raw fillet predicted by the Bayesian models were further compared with nutrient reference values (*NRV*_*s*_) for each essential element (*NRV*_*ee*_). The *NRVs* were defined as the daily reference values for adults from food to consumer under EU Regulation 1169/2011 [37]: 800 mg (calcium), 14 mg (iron), 150 μg (iodine), 55 μg (selenium), and 10 mg (zinc). According to this EU regulation, 100 g of food (e.g., fish fillets) can be regarded as a significant source of an essential element if it provides 15% or more of the *NRV*_*ee*_.

We also extracted published model-predicted concentrations of the sum of EPA (eicosapentaenoic acid) and DHA (docosahexaenoic acid) [38], and the sum of dioxin and dl-PCBs (dioxin-like polychlorinated biphenyls) [33] for selected species from the NEAO (Table S3b). The WHO 2005 toxic equivalency factors (TEF) were used to calculate the total Toxic Equivalent (TEQ) for dioxin + dl-PCBs here [39]. Due to missing data for certain nutrients in the models, we obtained the following: calcium of haddock (11 mg/100 g), EPA + DHA of Atlantic cod (229 mg/100 g [mean of median values of 2010, 2020, 2021, and 2022]) and haddock (0.2 g/100 g). Concentrations of all selected nutrients and contaminants (*e*) are provided in Table S3b.

### 2.2 Nutrients, contaminants, and global fish trade

We extracted bilateral trade mass data for the selected fish species (*s*) from the UN Comtrade Database [40] compiled by Chatham House Resource Trade Database (CHRTD) [41] based on the Harmonized Commodity Description and Coding System (HS) with commodities including whole frozen, fresh, or chilled fish (combined dataset in Table S4a). The mass trade by species (*TM*_*s*_, t) between exporters and importers were predicted using validated food trade models [25]. For this study, an exporter was included if: 1) the exporter captured these species in relevant ocean basins listed in the International Council for the Exploration of the Sea (ICES) catch datasets, and 2) the exporter captured ≥50% of the annual national fish catch biomasses from the NEAO. Hence, exporters included Denmark, Faroe Islands, Iceland, Ireland, Norway, Russian Federation, and United Kingdom, and importers included all possible countries and regions.

The traded whole fish masses were further converted to traded fillet masses and then traded nutrient and contaminant masses (*TEM*_*s,e*_, kg) using Eq. 1.

(1) $${TEM}_{s,e}={TM}_{s}\times \gamma_{s}\times C_{s,e}$$

**Fig. 1:**
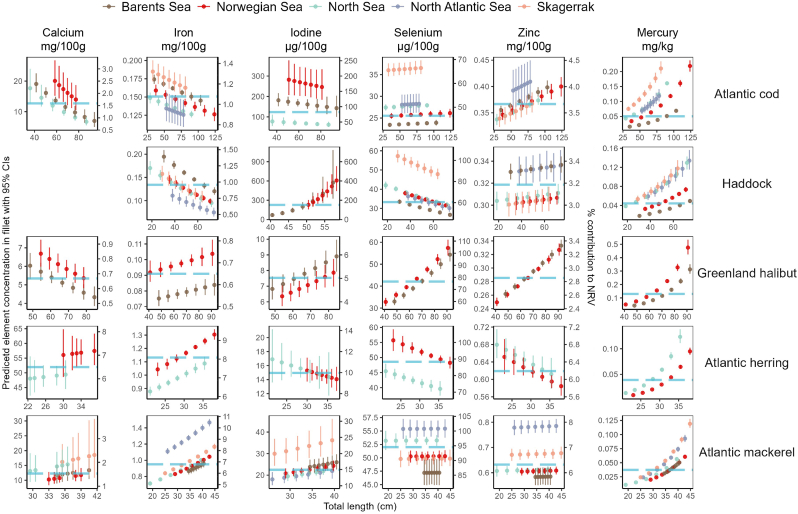
Model-predicted element concentrations (median and 95% confidence intervals, wet weight) for different total lengths and ocean basins. The secondary (right) axis indicates the percentage (%) contribution to relevant EU Nutrient Reference Value (NRV) for each essential element. The NRVs are defined under EU Regulation 1169/2011 [37]. Turquoise dashed line indicates the model predicted overall geometric mean element concentration. The range of total length was based on an existing study [30] and the intervals were evenly distributed within the range.

Where *γ*_*s*_ indicates the fillet to total weight ratio: 0.34 (Atlantic cod), 0.35 (haddock), 0.34 (Greenland halibut), 0.46 (Atlantic herring), and 0.54 (Atlantic mackerel) [42]; and *C*_*s,e*_ refers to model-predicted geometric mean element concentrations (mg/kg; Fig. 1) or nutrient and contaminant concentrations from external sources (Table S3b). The traded essential element trades in numbers of NRVs (${Tn}_{NRVs-s,ee}$) were calculated following Eq. 2a, and the same (${Tn}_{AIs-s,EPA+DHA}$) was used to calculate the traded EPA + DHA in number of AIs (adequate intake; 250 mg [43]) following Eq. 2b.

(2a) $${Tn}_{NRVs-s,ee}=\frac{TEM_{s,ee}}{{NRV}_{ee}}$$

(2b) $${Tn}_{AIs-s,EPA+DHA}=\frac{TEM_{s,EPA+DHA}}{{AI}_{EPA+DHA}}$$

### 2.3 Fish import variation and human consumption

Inevitably, apart from direct human consumption, imported fish are often used for fish/livestock feed, further production for export, and other non-human-food uses (e.g., pet food and supplement). Thus, we further adjusted the traded nutrient and contaminant masses to the portion for direct domestic human consumption only.

To calculate the average annual percentage (Γ) of the masses between import for domestic human food and total import, we used two FAO Food Balance Sheet (FBS) datasets: FishStatJ and FAOSTAT (Table S5).

Firstly, we extracted live-weight fish biomass data from the FAO FishStatJ FBS [44]. Food items in FBS are not species-based but categorised by FAOSTAT as “Pelagic fish” and “Demersal fish”. Thus, the selected species were grouped under each category: Atlantic cod, haddock, and Greenland halibut were under “Demersal fish”, while Atlantic herring and mackerel were under “Pelagic fish”. We pooled FBS data during 2010–2019 to match with other datasets in sequential analyses (data of 2020 was not available when data extraction was performed). The mass balance equation in the FishStatJ FBS (Eq. 3) contains masses (kg) of production (P), food imports (FIs), food exports (FEs), total food supply (TFS), non-food uses (NFUs), and stock variations (SVs).

(3) P+FIs=FEs+TFS+NFUs+SVs

FIs does not represent the total fish import due to the exclusion of quantities of fish used for meal reduction and other non-food uses (personal communication with FAO Fishery). Many importers import large quantities of fish (especially small pelagic species) for fish meal in their aquaculture production, e.g., Norway [45]. Therefore, we extracted total fish import (I) from the FAOSTAT FBS which included both edible and non-edible commodities to accurately link to the trade data. And Γ represents the percentage of TFS from FIs (${TFS}_{from\;FIs}$) relative to I (Eq. 4).

(4) $$\Gamma =\sum_{2010}^{2019}\frac{{TFS}_{from\;FIs}}{I}÷10\times 100\%$$

When the value of FIs was equal to or slightly smaller than the value of I, it suggested zero or little import of fish for non-food uses. When the I value was missing or when FIs was greater than I (likely due to the rounding down of numbers in FAOSTAT or possible human error when the difference was too big), we replace the FIs value with I.

To calculate the associated uncertainty of Γ, we estimated the range of Γ, including a lower bound ($\Gamma_{LB}$), middle bound ($\Gamma_{MB}$), and upper bound ($\Gamma_{UB}$) value. $\Gamma_{LB}$ represents the lowest possible value where TFS was primarily from P (Eq. 5). $\Gamma_{UB}$ represents the highest possible value where TFS was primarily from I (Eq. 6).

(5) $$\Gamma_{LB}=\left\{ \begin{array}{ll} 0 & P\geq TFS\\ \frac{TFS-P}{I}\times 100\% & P\;<\;TFS \end{array}\right.$$

(6) $$\Gamma_{UB}=\left\{ \begin{array}{ll} \frac{TFS}{I}\times 100\% & \;FIs\geq TFS\\ 100\% & \;FIs\;<\;TFS,I\leq TFS\\ \frac{FIs}{I}\times 100\% & \;FIs\;<\;TFS,I\;>\;TFS \end{array}\right.$$

$\Gamma_{MB}$ represents the scenario where the proportion of FIs contributing to TFS (${TFS}_{from\;FIs}$) is equivalent to the proportion of P contributing to TFS (${TFS}_{from\;P}$) (Eq. 7).

(7) $$\frac{{TFS}_{from\;FIs}}{FIs}=\frac{{TFS}_{from\;P}}{P}$$

Thus, $\Gamma_{MB}$ is calculated as:

(8) $$\Gamma_{MB}=\frac{TFS\times FIs}{\left(FIs+P\right)\times I}\times 100\%$$

Where, $TFS={TFS}_{from\;FIs}+{TFS}_{from\;P.}\;SVs$ can be negative, zero, and positive. When SVs is positive or zero, Eqs. 5, 6 and 8 remain the same. When SVs is negative, Eq. 6 remains the same but:

Eq. 5 is rewritten as $\Gamma_{LB}=\left\{ \begin{array}{ll} 0 & P-SVs\geq TFS\\ \frac{F-P+SVs}{I}\times 100\% & P-SVs<TFS \end{array}\right.$ , and

Eq. 8 is rewritten as $\Gamma_{MB}=\left(TFS+\frac{SV\times TFS}{FEs+NFU+TFS}\right)\times \frac{FIs}{\left(FIs+P\right)\times I}\times 100\%$ .

### 2.4 Global fish trade and human health indicators

To understand the potential nutritional benefits and health risks from mercury exposure of traded elements to the domestic population of the importer, we extracted 2018 population structure data (${Pop}_{age}$; existing data [46] and additional data for Andorra, American Samoa, Bermuda, Dominica, Eritrea, Faroe Islands, Greenland, Liechtenstein, Marshall Islands, Monaco, Nauru, Palau, San Marino, and Tuvalu; Table S6a), average requirement values (${AR}_{ee}$ [38], mg/day per capita or μg/day per capita; Table S6a) for the selected essential elements, adequate intake for EPA + DHA (${AI}_{EPA+DHA}$, mg/day per capita), and daily per capita total mercury exposure data (${\mathrm{Exp}}_{Hg}$ [47], mg/day per capita; Table S6b). Based on these, we calculated the total annual domestic requirements (${TDR}_{e}$, kg/a) and total mercury exposure (${TE}_{Hg}$, kg/a), then the annual contribution (${Contri}_{e}$, %) from trade of all selected species to ${TDR}_{ee}$ or ${TE}_{Hg}$.

(9) $${TDR}_{e}=\sum_{age}\left({Pop}_{age}\times 365\times {AR}_{ee,age}\;\text{or}{\;AI}_{EPA+DHA}\;\right)$$

(10) $${TE}_{Hg}={Pop}_{total}\times 365\times {\mathrm{Exp}}_{Hg}$$

(11) $${Contri}_{e}=\sum_{Species}\frac{\sum_{2010}^{2020}{TEM}_{s,e}\times \Gamma /11}{{TDR}_{ee}\;\text{or}\;{TE}_{Hg}}\times 100\%$$

Where, ${Pop}_{total}$ indicates the total population. Similar to Γ, ${Contri}_{e}$ also has ${Contri}_{e,LB}$, ${Contri}_{e,MB}$, and ${Contri}_{e,UB}$.

### 2.5 Fish body size variation and element yields

To examine the effects of body size structure in fish catch on total element yields, we extracted annual fish catch data with weight at age and catch in numbers by age from 2010 to 2020 based on ICES working group reports (AFWG 2021 [48], WGNSSK 2021 [49], WGWIDE 2021 [50], and HAWG 2021 [51]) for the major stocks (*st*) of Atlantic cod, haddock, Atlantic herring, and Atlantic mackerel (Table S7). For each stock, catch biomass by age ($B_{st,age}$, t) was calculated as the product of the whole fish weight at age ($w_{st,age}$, g) and catch in numbers by age ($n_{st,age}$) (Eq. 12).

(12) $$B_{st,age}=w_{st,age}\times n_{st,age}$$

For a specific age, $w_{st,age}$ was converted to total length at age ($L_{st,age}$, cm) using the established total length to weight relationships [52] following Eq. 13.

(13) $$w_{st,age}=a\times L_{st,age}^{b}$$

Where, the conversion coefficients are *a* = 0.0069 and *b* = 3.08 (Atlantic cod), *a* = 0.0059 and *b* = 3.13 (haddock), *a* = 0.0059 and *b* = 3.09 (Atlantic herring), and *a* = 0.0035 and *b* = 3.26 (Atlantic mackerel).

Hence, the function of the catch biomass to total length category can be written as:

(14) $$B_{st,age}=a\times L_{st,age}^{b}\times n_{st,age}$$

Where, the total length at age category was calculated from the *w*_*st*__,__*age*_ described by Eq. 13 as:

(15) $$L_{st,age}=\sqrt[{b}]{\frac{w_{st,age}}{a}}$$

Each selected stock was assigned to one or more ocean basins (Table S3c), and the element concentrations by total length category were predicted assuming the fat content (if available), sea temperature, and salinity to be the species-mean of the associated ocean basin(s). Although the catch data are linked to specific fish stocks and certain fish species can migrate across ocean basins, we fixed the ocean basin of each stock based on their primary habitat (e.g., Northeast Arctic cod was assigned to the Barents Sea even if some individuals migrate to the Norwegian Sea for spawning). For stocks from multiple ocean basins, the mean concentrations from all ocean basins were calculated. Thus, for each stock, the annual element yields by total length category ($M_{st,e,L}$, t) were calculated as:

(16) $$M_{st,e,L}=B_{st,age}\times C_{s,e,L}$$

Where, $C_{s,e,L}$ (mg/kg) is the model-predicted geometric mean element concentrations by species/stock, element, and total length category (Table S3c).

The annual total element yields for each stock were then calculated as:

(17) $$M_{st,e}=\sum_{L}M_{st,e,L}$$

And the annual catch-biomass-weighted-average element concentrations for each stock (${\bar{C}}_{st,e}$, mg/kg) were calculated as:

(18) $${\bar{C}}_{st,e}=\frac{M_{st,e}}{\sum_{age}B_{st,age}}$$

All data analyses were conducted in R (Version 3.6.0) and operated in RStudio (Version 1.1.453).

## 3 Results

### 3.1 Trade of fish nutrients and contaminants

During 2010−2020, catch from NEAO fisheries were exported to 155 countries/regions. For Atlantic cod, haddock, Greenland halibut, and Atlantic herring, the top three exporters by biomass were Norway, the Russian Federation, and Denmark; whereas for Atlantic mackerel, the top three exporters were Norway, the United Kingdom, and Iceland. The main importer of these fish was the Chinese mainland, followed by the Russian Federation, Japan, Germany, Denmark, the Netherlands, the United Kingdom, and Vietnam (Table 1, Table 2, Fig. S1).

**Table 1:** Accumulative trades of fish (2010−2020) from the top three exporting and importing countries/regions, expressed in million tonnes (Mt) of fillet weight.

Species	Exporter	Export biomass	Importer	Import biomass
Atlantic cod (Mt)	Russian Federation	0.503	Chinese mainland	0.344
	Norway	0.427	Netherlands	0.122
	Denmark	0.148	Denmark	0.108
*Total*	*1.17 Mt*			

Haddock (Mt)	Norway	0.251	Chinese mainland	0.138
	Russian Federation	0.072	United Kingdom	0.117
	Denmark	0.029	Denmark	0.032
*Total*	*0.402 Mt*			

Greenland halibut (Mt)	Denmark	0.099	Chinese mainland	0.073
	Norway	0.055	Vietnam	0.042
	Russian Federation	0.046	Japan	0.033
*Total*	*0.253 Mt*			

Atlantic herring (Mt)	Norway	0.975	Chinese mainland	0.645
	Russian Federation	0.862	Russian Federation	0.371
	Denmark	0.364	Germany	0.281
*Total*	*3.00 Mt*			

Atlantic mackerel (Mt)	Norway	1.70	Chinese mainland	0.514
	United Kingdom	0.645	Russian Federation	0.435
	Iceland	0.525	Japan	0.398
*Total*	*4.15 Mt*			

Note: Trade includes all uses (food and non-food). Fish product included frozen, fresh, and chilled whole fish.

**Table 2:** Accumulative trades of important nutrients and contaminants associated with fish fillets (2010–2020) from the top three exporting and importing countries/regions.

Nutrient/Contaminant	Exporter	Export	Importer	Import
Calcium (NRV eq.)	Norway	1.00 billion	Chinese mainland	576 million
	Russian Federation	682 million	Russian Federation	308 million
	United Kingdom	311 million	Germany	197 million
*Total*	*2.84 billion NRV eq.*			

Iron (NRV eq.)	Norway	2.02 billion	Chinese mainland	925 million
	Russian Federation	891 million	Russian Federation	596 million
	United Kingdom	699 million	Nigeria	414 million
*Total*	*5.43 billion NRV eq.*			

Iodine (NRV eq.)	Norway	10.9 billion	Chinese mainland	6.37 billion
	Russian Federation	6.40 billion	United Kingdom	2.52 billion
	Denmark	2.42 billion	Netherlands	1.89 billion
*Total*	*25.1 billion NRV eq.*			

Selenium (NRV eq.)	Norway	28.6 billion	Chinese mainland	13.5 billion
	Russian Federation	12.6 billion	Russian Federation	7.42 billion
	United Kingdom	9.11 billion	Nigeria	5.29 billion
*Total*	*75.6 billion NRV eq.*			

Zinc (NRV eq.)	Norway	1.93 billion	Chinese mainland	915 million
	Russian Federation	875 million	Russian Federation	506 million
	United Kingdom	618 million	Nigeria	359 million
*Total*	*5.11 billion NRV eq.*			

EPA + DHA (AI eq.)	Norway	369 billion	Chinese mainland	149 billion
	United Kingdom	133 billion	Russian Federation	104 billion
	Russian Federation	120 billion	Nigeria	78.4 billion
*Total*	*964 billion AI eq.*			

Mercury (kg)	Norway	141	Chinese mainland	77.3
	Russian Federation	75.4	Russian Federation	31.0
	Denmark	44.5	Netherlands	24.3
*Total*	*382 kg*			

Dioxin + dioxin-like PCBs (mg TEQ)	Norway	254	Chinese mainland	128
	Russian Federation	111	Russian Federation	71.0
	Denmark	86.9	Japan	50.8
*Total*	*715 mg TEQ*			

Note: Values are expressed as nutrient reference value (NRV) equivalents for calcium, iron, iodine, selenium, and zinc; average intakes (AI) equivalent for EPA + DHA; kg for mercury; and mg of total toxic equivalent (TEQ) for dioxin + dioxin-like PCBs. eq., equivalent.

**Fig. 2:**
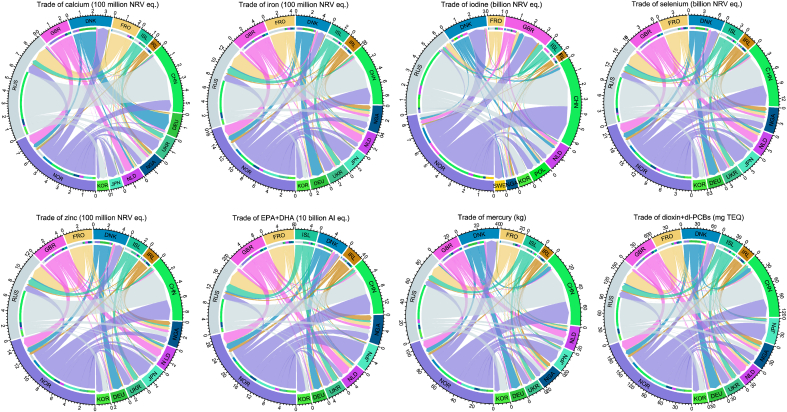
Accumulative trade of important nutrients and contaminants in fillet via NEAO fish trade during 2010–2020 from relevant exporters to top 10 importers. For importer ISO3 code, see Table S4a.

From these trade pathways, 2.84 billion NRV equivalents of calcium, 5.43 billion NRV equivalents of iron, 25.1 billion NRV equivalents of iodine, 75.6 billion NRV equivalents of selenium, 5.11 billion NRV equivalents of zinc, 964 billion AI equivalents of EPA + DHA, 382 kg of mercury, and 715 mg TEQ of dioxin + dl-PCBs were traded globally regardless of use (Tables 2 and S4, Figs. 2 and S2). Norway was the main exporter by mass of all nutrients and contaminants, followed by the Russian Federation except for EPA + DHA. The United Kingdom ranked the 2nd exporter for EPA + DHA and the 3rd for calcium, iron, selenium, and zinc, while Denmark ranked 3rd for iodine, mercury, and dioxin + dl-PCBs. The Chinese mainland was the main importer by mass of all nutrients and contaminants, followed by the Russian Federation (except for iodine) and the United Kingdom (iodine). Germany, Nigeria, the Netherlands, and Japan ranked as the third-highest importers, with rankings varying between nutrients and contaminants.

### 3.2 Fish trade, intake, and exposure rates

The middle-bound percentage of imported fish for direct domestic human consumption ($\Gamma_{MB}$) differed greatly between fish groups and importers (Table S8). For demersal fish, $\Gamma_{MB,demersal}$ ranged from 0.3% (Falkland Islands [Malvinas]) to 100.0% (e.g., Bermuda, Chad, and South Sudan), while for pelagic fish, $\Gamma_{MB,pelagic}$ ranged from 0.4% (Norway) to 100.0% (Cayman Islands). The main fish importer, the Chinese mainland, had low $\Gamma_{MB,pelagic}$ (1.0%) but high $\Gamma_{MB,demersal}$ (82.2%).

**Fig. 3:**
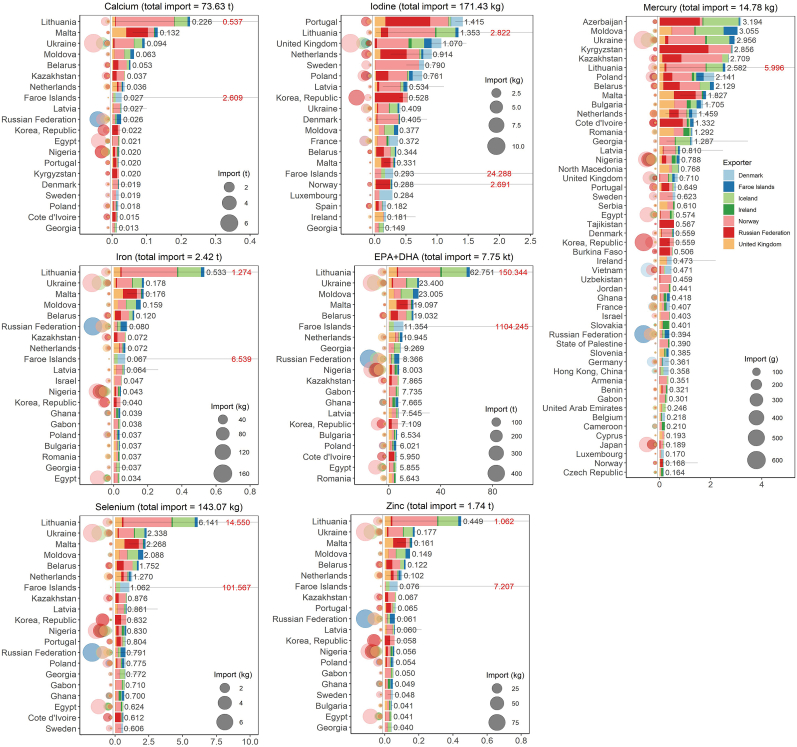
Mean annual contributions (Contri_e_, %) of important nutrients and mercury from imported fish fillets for direct human consumption to total annual domestic nutrient requirements and all-source mercury exposure. Top 20 importers (by Contri_e_) are shown for nutrients and top 50 for mercury. In each plot, mean annual total imported mass for direct human consumption is indicated on top, mean annual total imported mass for direct human consumption from each exporter is shown by the size of bubbles on the left, and Contri_e,MB_ values are plotted on the right. The Contri_e,LB_ and Contri_e,UB_ are shown (horizontal bar) and the Contri_e,UB_ value beyond the axis range is indicated in red.

Imported calcium, iron, and zinc contributed little to domestic intake (${Contri}_{e,MB}<1.00\%$), while iodine, selenium, and EPA + DHA had moderate to high contributions. The top three ${Contri}_{e,MB}$ were from Portugal (1.41%), Lithuania (1.35%), and the United Kingdom (1.07%) for iodine, Lithuania (6.14%), Ukraine (2.34%), and Malta (2.27%) for selenium, and Lithuania (62.75%), Ukraine (23.40%), and Moldova (23.00%) for EPA + DHA (Fig. 3, Table S9a). Among these top importers, only the United Kingdom (iodine) and Ukraine (selenium, EPA + DHA) imported large nutrient masses. In contrast, for the top nutrient importer, the Chinese mainland, the imported iodine, selenium, and EPA + DHA contributed little to domestic intake (0.12%, 0.06%, and 0.12%, respectively). For all importers, trade pathways contributed little to moderately to domestic total mercury exposure (${Contri}_{Hg,MB}\;<\;4\%$) with the top three values from Azerbaijan (3.19%), Moldova (3.06%), and Ukraine (2.96%), among which only Ukraine had high mercury import (Fig. 3, Table S9b). Additionally, the top three upper-bound contributions to mercury exposure (${Contri}_{Hg,UB}$) were Lithuania (6.00%), Georgia (3.46%), and Ukraine (3.21%). The main Hg importer, the Chinese mainland had very low ${Contri}_{Hg,MB}$ (0.16%) and ${Contri}_{Hg,UB}$ (0.41%).

### 3.3 Body size, nutrient yields, and contaminant exposure

Predicted mercury concentrations increased exponentially with fish length, whereas changes in essential elements were relatively linear (Fig. 1). Fish length did not determine which species were significant sources (contributing >15.0% NRV for 100 g fillet [standard portion hereafter], see Section 2.1), except for Atlantic mackerel where individuals <32 cm from the Norwegian Sea were not significant sources of iodine. Contributions to NRV (%NRV) with increasing length for some species doubled (e.g., iodine in Barents Sea haddock: %*NRV*_*I*,51.8__cm_ = 180.0% [95% CIs = 136.0%–242.0%] and %*NRV*_*I*,58.3__cm_ = 405.0% [294.0%–553.0%]), some halved (e.g., calcium in North Sea Atlantic cod: %*NRV*_*Ca*,36.0__cm_ = 2.2% [1.5%–3.0%] and %*NRV*_*Ca*,87.0__cm_ = 0.8% [0.7 %–1.0%]), while some remained unchanged (e.g., selenium in Atlantic mackerel). For large-sized haddock (>50 cm), the iodine yields in a standard portion were likely above the tolerable upper intake limit for children and adolescents aged 1−17 years (200−500 μg/day).

Regardless of length, all species were significant sources of selenium but not calcium, iron, or zinc, and Atlantic cod and haddock were significant sources of iodine (Fig. 1). With a standard portion, Atlantic cod, haddock, and Greenland halibut contributed less than 4.0% NRVs for calcium, iron, and zinc. For Atlantic herring and mackerel, %NRV values were higher, but still <10.0%.

Within the investigated length ranges, model-predicted mercury concentrations were lower than the current maximum level (ML) values under EU Regulation 2023/915 [53] (Fig. 1), except for larger-sized Greenland halibut (approximately 90 cm) from the Norwegian Sea that might exceed the ML (0.5 mg/kg wet weight). As a result, consuming one standard portion per week of any investigated species within the investigated length ranges will likely not exceed the tolerable weekly intake (TWI) for methyl mercury (1.3 μg/kg body weight [54], assuming 70 kg of body weight and that all mercury is in the methylated form).

**Fig. 4:**
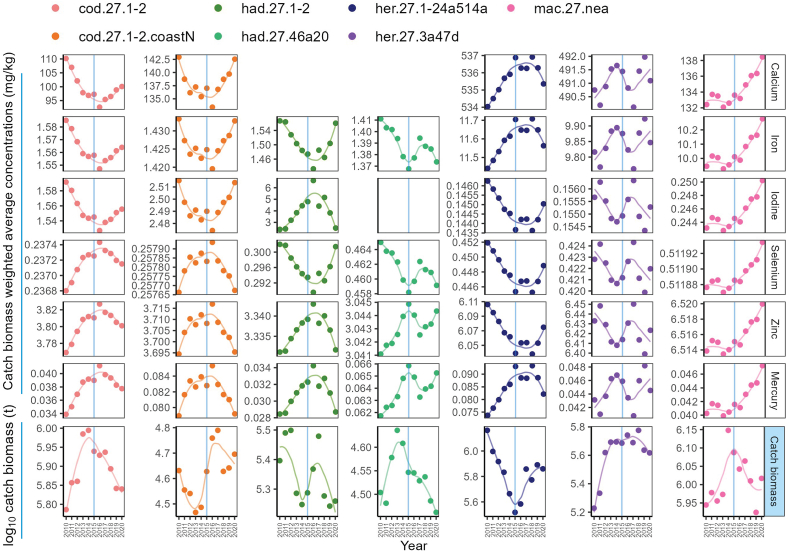
Catch biomass weighted element concentrations (mg/kg wet weight) and annual total catch biomass (t) of major stocks of Atlantic cod, Atlantic herring, Atlantic mackerel, and haddock in the Northeast Atlantic Ocean between 2010 and 2020. Locally weighted regression (Loess) was applied to visualise the annual trend. The blue vertical line indicates the year 2015. For the stock code, cod.27.1-2: Northeast Arctic cod; cod.27.1-2.coastN: Northern Norwegian coastal cod; had.27.1-2: Northeast Arctic haddock; had.27.46a20: haddock from North Sea, West of Scotland, and Skagerrak; her.27.1-24a514a: Norwegian spring-spawning herring; her.27.3a47d: autumn spawning herring from North Sea, Skagerrak, Kattegat, and Eastern English Channel; and mac.27.nea: mackerel from the Northeast Atlantic and adjacent waters.

Total catch biomass of major Northeast Atlantic fish stocks changed between 2010 and 2020 (Fig. 4); and this was also the case for the weighted average concentrations of all elements and species. The greatest change in estimated concentrations was iodine in haddock and the concentration during 2016 (6.62 mg/kg) was more than twice the concentration in 2010–2011 (2.39−3.19 mg/kg), reflecting significant annual variation.

## 4 Discussion

The NEAO fish trade pathways globalised high amounts of nutrients, including iodine, selenium, EPA + DHA. Although domestic direct consumption of traded fish varied among 155 importer countries/regions, these trade pathways contributed significantly to annual domestic EPA + DHA requirements for small-population importers (e.g., Lithuania) but not for high-population importers (e.g., the Chinese mainland). Traded amounts of mercury, dioxin + dl-PCBs from the NEAO fish were low, and associated pathway contributions to total domestic mercury exposures were <4%. Changes in fish body size affected nutrient and contaminant fillet concentrations and subsequently trade dynamics of nutrients and contaminants. Our study provides valuable insights regarding seafood globalization and marine fish trade that can be used to support adaptive management strategies for contaminants as well as nutrition-sensitive policies. Overall, our investigation reports that trade is important for modulating nutrition in several countries/regions and that overall mercury and dioxin + dl-PCBs contaminant exposure rates were low.

Top producers of aquatic foods (e.g., Chinese mainland and Norway [55]) import pelagic fish largely for feed (Table S8) to produce higher sized/valued aquaculture species. While many of the nutrients will be consumed, nutrient loss is expected through inefficient conversion of feed to fish, limiting use of edible nutrients in wild-caught fishes [45]. This suggests that pelagic species including Atlantic herring and mackerel can be more effectively utilized for human health which may be achieved through policies aimed at improving direct consumption (e.g., food literacy, local health and nutrition education) [56]. Additionally, because micronutrient deficiencies are common among exporters (e.g., iodine and iron [46]), utilising more local production for domestic consumption may help manage these deficiencies [57]. However, challenges remain due to domestic production [3], affordability, decreases in consumption, and collapses of local marine fisheries in some countries/regions [58].

The estimated contributions of fish trade to domestic intake and exposure (${Contri}_{e}$) were governed by total population size and demography compared to import mass or catch composition. Top importers such as the Chinese mainland and Nigeria had low ${Contri}_{e}$ due to the dilution effect of large human population sizes whereas importers with low populations (Lithuania, Portugal) had high ${Contri}_{e}$ despite lower traded nutrient masses (e.g., ${Contri}_{Se,MB}=3.6\%$ and ${Contri}_{EPA+DHA,MB}=32.6\%$ [Norway−Lithuania], ${Contri}_{I,MB}=0.7\%$ [Russian Federation−Portugal]; Fig. 3, Table S9a). For importers with small populations, fish trade greatly supported essential nutrient intake, which was not the case for highly populated importers. Besides, for some highly populated countries/regions with high production, micronutrient deficiencies were still prevalent (e.g., India [59,60]). While their total fish import from NEAO catch (10.8 t of fillet from the selected species between 2010 and 2020; Table S4) and export-production ratio of fish were low (*FEs*/*P* ≈ 15% per year; Table S5), increasing import and improving nutritional quality of fish feed thus increasing concentrations of nutrients in fish fillets [61,62] could be implemented. Another way to improve essential nutrient intake is to diversify use of non-fillet fish parts. For example, Nigeria allocated fish import mainly for direct consumption ($\Gamma_{MB,demersal,Nigeria}=94.9\%$ and $\Gamma_{MB,pelagic,Nigeria}=80.3\%$; Table S8). Nevertheless, they also imported cod heads ([63], Table S10) which provide another vital source of several micronutrients and macronutrients [64]. Such practice can further improve essential nutrient intake, while simultaneously reducing food waste in support of regional and global sustainability [65].

Landlocked countries/regions relied on marine fish trade from the NEAO catch to improve nutrient intake (e.g., Moldova, Belarus, and Kyrgyzstan; Fig. 3, Table S9a) even when some had low per capita fish/seafood consumption (FAO Our World in Data). These supply chains are important and should be secured. For these importers with both low aquatic food production and micronutrient deficiencies, global fish trade appears to fail to deliver essential nutrients to those most in need [1]. For example, between 2010 and 2020, Cambodia only imported 0.23 t of whole Atlantic mackerel from Norway in 2017 (Table S4) which contributed little to domestic iodine requirements (Table S9a). Although Cambodia also imports aquatic foods from neighbouring exporters (e.g., Thailand and Vietnam; see relevant CHRTD data [41]), iodine deficiency is still prevalent. Increasing imports of iodine-dense fish could reduce deficiencies, however, would require nutrition-sensitive trade policies and international aid [1], and the trade-offs between the carbon footprints of associated species [2,66] and nutritional gain should not be overlooked.

Low to moderate mercury risks were found within trade pathways from the NEAO, even under worst-case scenarios (i.e., ${Contri}_{Hg,UB}$; Fig. 3). This was also the case when compared to exposure from total marine fish consumption (e.g., ${Contri}_{Hg,UB}=6.00\%$ [all exporters-Lithuania]; Table S9b) or when considering potential economic losses [47]. Projected global anthropogenic mercury emissions may increase under certain scenarios (e.g., business as usual) potentially affecting human consumers [47]. However, all NEAO fish that we analysed had Se:Hg molar ratios >1 (Fig. S3) which suggests a positive health effect [9,10]. However, some limitations exist in this approach especially where human data is scarce, and further research into Se−Hg interactions human health impacts is required. The estimated uncertainty levels (i.e., LB, MB, and UB) of the nutrient contributions and mercury exposure from fish trade were much greater for some importers (e.g., Faroe Islands; Fig. 3), which is likely a result of a combination of low population, high import and domestic production (Table S5).

Global fish trade also serves as an important source of persistent organic pollutants (POPs) including PCB-153 [25]. In the present study, the total traded 715 mg TEQ of dioxin + dl-PCBs is equivalent to 357.5 trillion TWIs (2 pg/kg body weight [67]), which is slightly higher than traded mercury (293.8 trillion TWIs). The associated contribution to the relevant TWI is low for all importers regardless of population. For example, the highest estimated annual exposure per capita for the importers from the investigated trade pathways was 161 pg TEQ (Lithuania; Table S9c). This translates into 0.0063 pg TEQ/kg body weight per day (assuming 70 kg body weight), which is significantly lower than the mean estimated upper bound exposure in Europe (i.e., 0.4−2.6 pg TEQ/kg body weight per day [67]). However, no relevant human exposure data exists, thus, contribution to human exposures of these compounds via global fish trade could not be estimated.

Our study also highlights the importance of accounting for fish size within nutrition-sensitive approaches to food and environmental policies. The size-specific nutrient and contaminant concentrations (Fig. 4) affected the nutrient yields from catch (Fig. S4) and possibly the yields via fish trade. Between 2011 and 2016, haddock export from Norway to the Chinese mainland decreased from 12.3 kt to 7.1 kt (Table S4a). However, the associated iodine yield increased from 30.1 kg to 47.0 kg when we applied weighted average concentrations to more accurately estimate nutrient yields. Also, the often-coarse grouping of “fish/seafood” masks variation of nutrient/contaminant concentrations among species, leading to inaccuracies regarding actual intake of certain nutrients and exposure to hazards, thus providing misleading information regarding risks and benefits. Apart from population-level implications, fish body size and geography of the ocean basin (e.g., origin) may also affect nutrient yields and contaminant exposures for a given portion size of fillet (100 g; Fig. 1). This can be particularly vital for specific vulnerable groups. For example, a standard portion of fillet from bigger sized haddock can provide a good amount of iodine (Fig. 1), which is particularly beneficial for population groups with high intake requirements (e.g., pregnant or lactating women; National Institutes of Health). However, the predicted fillet iodine concentrations of bigger haddock (Fig. 1) and the weighted average fillet iodine concentrations of haddock caught in the NEAO in certain years (Fig. 4), when converted to a standard portion, were quite close to the maximum upper levels for young children (National Institutes of Health). While this could be a potential concern [68], the risk of surpassing the upper levels remains low, since fish intake in children is generally low [69,70]. Such size- and species-variability should be communicated in dietary guidelines for fish/seafood, such as targeted advice for susceptible population groups (e.g., in Norway, pregnant women are advised to not consume Greenland halibut weighing more than 3 kg). Additionally, nutritional importance of fish should be more recognised in national dietary guidelines and although the concentrations of some essential elements may be low, they are comparable to other dietary sources [71] and may enhance the bioavailability of some nutrients [72].

Current fishery regulations and policies often do not consider nutrition, however, the maximum nutrient yield theory [18] advocates for combining different fish species to maximize nutrient yields from fish catches, and our study suggests that including fish size in relevant discussion can further improve nutrient yields. In response to the body-size-shrinking [36,73], earlier-matured [74,75], yet stable-biomassed [36] global fish assemblages due to both climate change and anthropogenic impacts, we may adopt a more balanced exploitation pattern, where catches are proportional to size-based production [76,77]. While climate change is predicted to also affect global aquatic food systems in terms of nutrients [78,79], consuming smaller individuals may counteract the nutrient loss in the NEAO fisheries based on our findings. In the Barents Sea, with a warming trend in seawater over the past decades [80], smaller-sized individuals remained higher in certain nutrients and lower in mercury for all species compared with the bigger ones (Fig. 1), suggesting that the additive effects on improved nutrient intakes and reduced mercury exposures may manifest when harvesting smaller individuals. Overall, nutrient yields and fish body size should be considered in the context of human health, seafood safety, and sustainable fisheries, and how these may respond to a rapidly changing climate.

To improve the yield of EPA + DHA and other n-3 polyunsaturated fatty acids (n-3 PUFA), seasonality plays an important role and should be considered especially for pelagic fish [38]. Additionally, as concentrations of POPs can be high in fish with high fat content [33], trade-offs between the risks of POPs exposures and benefits of n-3 PUFA in fisheries and global trade should be considered.

## 5 Study limitations

We acknowledge that there are some limitations in our investigation. First, there were likely other important drivers affecting element concentrations that were not included in the predictive models that need to be reconciled including spatial scale differences [81], oceanographic conditions, biogeochemical cycling [82], and population-specific variation [30,83]. Second, trade data from different landing areas were not available, thus area-specific concentrations were not used here. Third, the FAOSTAT fish groups (i.e., pelagic and demersal) can lead to uncertainty in species-level Γ estimation since small pelagic species are more likely to be processed as feed [84] while large pelagic species are often consumed directly [85]. Fourth, we assumed that the allocation of elements to human consumers perfectly matched the nutrient average requirements (ARs) and adequate intakes (AIs), as well as Hg exposure, but this is often not the case due to unequal distributions of seafood to different population groups [86]. Fifth, apart from Hg, dioxin, and dl-PCBs, other emerging contaminants including microplastics (MP) and per- and polyfluoroalkyl substances (PFAS), which are also found in marine fishes, can pose a health threat to human consumers [87,88]. However, they are not included in the present study due to a lack of predictive models for MP or PFAS in NEAO fish. Also, a limitation of our analysis is that it does not consider temporal scale, and investigating global fish trade and associated analyses over time would likely provide valuable insights. Finally, when estimating stock-specific, size-based element yields, we applied the model-predicted mean concentrations due to inaccessibility of finer-resolution data (e.g., time and location of catch) and assumed the same length to weight relationship within species while growth rates likely vary substantially between stocks and across geographical areas [89].

## 6 Conclusions

Our study provides a novel approach to estimate risks and benefits of global fish trade on domestic populations by tracking nutrients and contaminants from the ocean to human consumers. NEAO fisheries are important for global food and nutritional security, however, the estimated contributions of nutrient intakes and contaminant exposures vary across countries/regions. We recommend to adopt nutrition-sensitive fish trade to prioritise the import of certain nutrient-dense fish species and use traditional nutritious fish for feed as human food to address malnutrition. Moreover, we recommend that more detailed nutritional benefits of fish and risks and benefits of fish consumption in relation to fish body size should be considered in public policy making (e.g., food trade, fishery regulations, national and regional dietary guidelines) for better and safer nutrient yields among global populations and to tackle relevant issues resulted from the decreasing resilience of the global food trade systems [90]. NEAO fisheries only represents a fraction of the world’s aquatic food production, our analyses can be refined and scaled up to a global level to further examine trade pathways and be incorporated into future global food trade system analyses. This requires a more thorough understanding of regional nutrient concentration variation, species-based bilateral trade information (e.g., ARTIS database [4]), local/domestic trade information [25], and higher resolution national/regional food balance data.

## CRediT authorship contribution statement

**Yiou Zhu:** Writing – review & editing, Writing – original draft, Visualization, Validation, Methodology, Investigation, Formal analysis, Data curation, Conceptualization. **Quang Tri Ho:** Writing – review & editing, Writing – original draft, Validation, Methodology, Investigation, Formal analysis, Data curation, Conceptualization. **James P.W. Robinson:** Writing – review & editing, Methodology, Investigation. **Marian Kjellevold:** Writing – review & editing, Writing – original draft, Methodology, Investigation. **Ruirong Chang:** Writing – review & editing, Methodology, Investigation, Formal analysis. **Edvin Fuglebakk:** Writing – review & editing, Methodology, Investigation, Formal analysis, Data curation. **Jianmin Ma:** Writing – review & editing, Methodology. **Shijie Song:** Writing – review & editing, Methodology. **Lisbeth Dahl:** Writing – review & editing, Writing – original draft, Investigation. **Ole Jakob Nøstbakken:** Writing – review & editing, Writing – original draft, Investigation, Conceptualization. **Maria W. Markhus:** Writing – review & editing, Writing – original draft, Investigation. **Bente M. Nilsen:** Writing – review & editing, Investigation. **Tanja Kögel:** Writing – review & editing, Investigation. **Anne-Katrine Lundebye:** Writing – original draft, Investigation. **Atabak M. Azad:** Writing – review & editing, Investigation. **Abimbola Uzomah:** Writing – review & editing, Investigation. **Jeppe Kolding:** Writing – review & editing, Writing – original draft. **Vidar S. Lien:** Writing – review & editing, Methodology, Investigation. **Martin Wiech:** Writing – review & editing, Investigation. **Yanxu Zhang:** Writing – review & editing, Methodology, Conceptualization. **Amund Maage:** Writing – review & editing, Supervision, Project administration, Funding acquisition, Conceptualization. **Livar Frøyland:** Writing – review & editing, Supervision, Project administration, Funding acquisition. **Michael S. Bank:** Writing – review & editing, Supervision, Project administration, Methodology, Investigation, Funding acquisition, Formal analysis, Conceptualization.

## Conflict of competing interest

Dr. Michael S. Bank is an Associate Editor at this journal and was not involved in the editorial review or the decision to publish this article. All other authors declare that they have no known competing financial interests or personal relationships that could have appeared to influence the work reported in this paper.
