# Supplementary material for: Global marine fish trade networks track international pathways of nutrients and contaminants
Source: Eco Environ Health. 2026 Jan 29;5(1):100218. doi: 10.1016/j.eehl.2026.100218 (PMC12914667; doi:10.1016/j.eehl.2026.100218)
Supplement: Multimedia component 1 [file mmc1.docx]

**Supplementary Materials**

Global marine fish trade networks track international pathways of nutrients and contaminants

**Author names:**

Yiou Zhu^a,*^, Quang Tri Ho^a^, James P.W. Robinson^b^, Marian Kjellevold^a^, Ruirong Chang^c^, Edvin Fuglebakk^a^, Jianmin Ma^d^, Shijie Song^e^, Lisbeth Dahl^a^, Ole Jakob Nøstbakken^a^, Maria W. Markhus^a^, Bente M. Nilsen^a^, Tanja Kögel^a^, Anne-Katrine Lundebye^a^, Atabak M. Azad^a^, Abimbola Uzomah^f^, Jeppe Kolding^g^, Vidar S. Lien^a^, Martin Wiech^a^, Yanxu Zhang^h^, Amund Maage^a^, Livar Frøyland^a^, Michael S. Bank^a,i,*^

**Affiliations:**

^a^ Institute of Marine Research, Bergen 5817, Norway

^b^ Lancaster Environment Centre, Lancaster University, Lancaster LA1 4YQ, United Kingdom

^c^ Joint International Research Laboratory of Atmospheric and Earth System Sciences, Nanjing University, Nanjing 210093, China

^d^ College of Urban and Environmental Sciences, Peking University, Beijing 100871, China

^e^ College of Earth and Environmental Sciences, Lanzhou University, Lanzhou 730000, China

^f^ Federal University of Technology, Owerri, PMB 1526, Nigeria

^g^ University of Bergen, Bergen 5020, Norway

^h^ Department of Earth and Environmental Sciences, Tulane University, New Orleans, LA 70118, U.S.

^i^ University of Massachusetts Amherst, Amherst, MA 01003, U.S.

***Corresponding authors.**

Email: [mikezhugreenerworld@gmail.com](mailto:mikezhugreenerworld@gmail.com) (Y. Zhu); [Michael.bank@hi.no](mailto:Michael.bank@hi.no) (M. Bank)

**Figures:**

See separate file for high resolution Fig. S1 (“Fig. S1_Zhu et al.tiff”).

Fig. S1. Accumulative global fish trade of NEAO catch in metric tonnes between 2010 and 2020. For ISO3 codes of trade partners, see Table S4a.

See separate file for high resolution Fig. S2 (“Fig. S2_Zhu et al.tiff”).

Fig. S2. Accumulative trade of masses of nutrients and contaminants of NEAO catch via global fish trade between 2010 and 2020. For ISO3 codes of trade partners, see Table S4a.


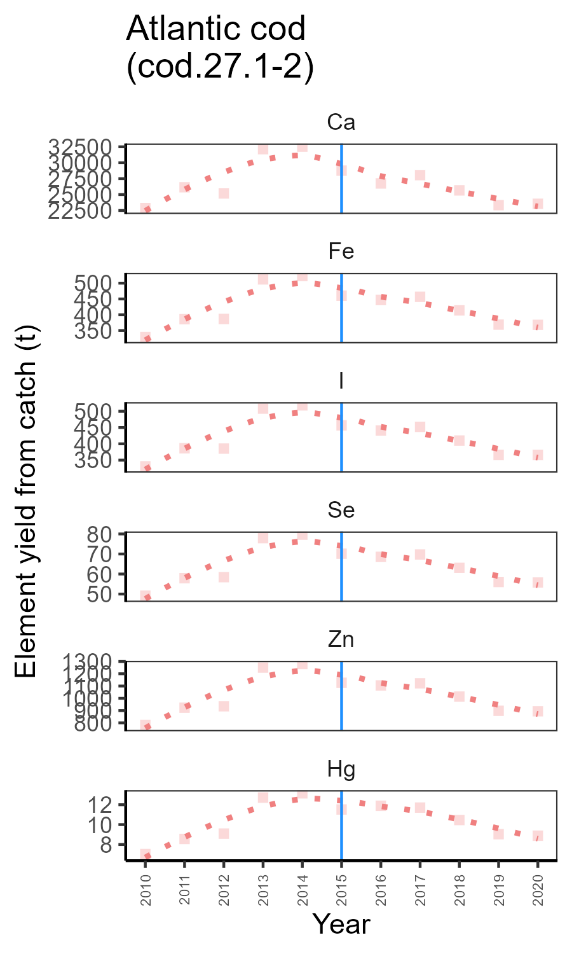

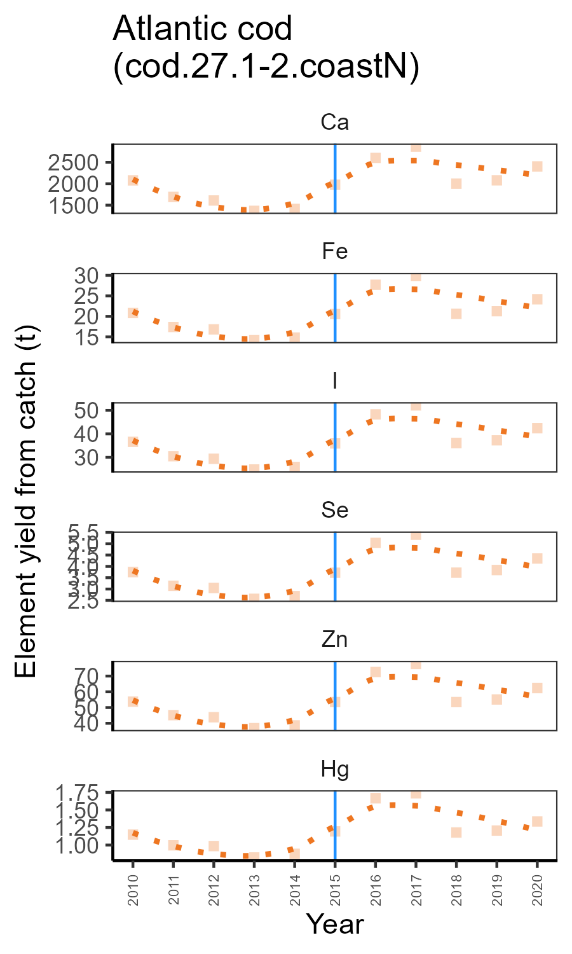


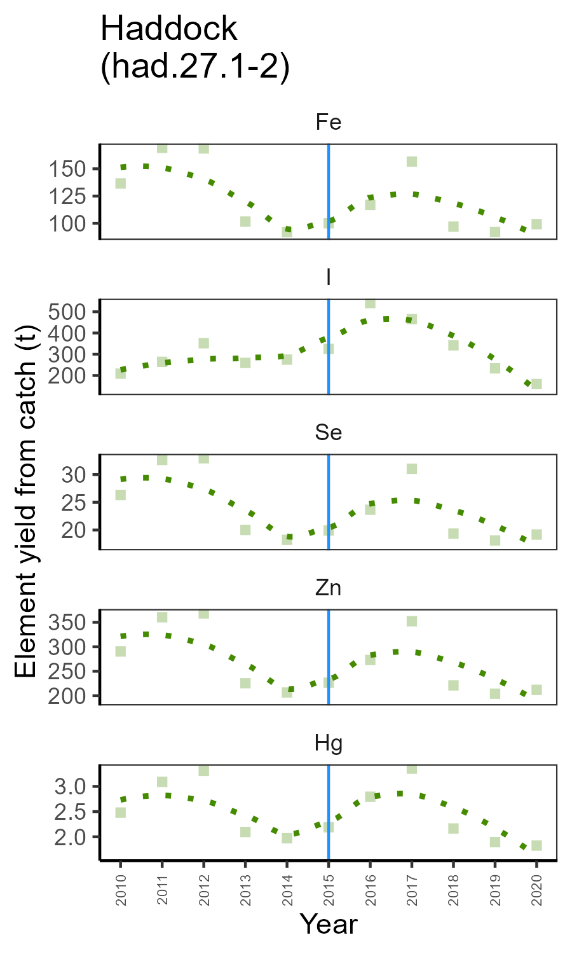

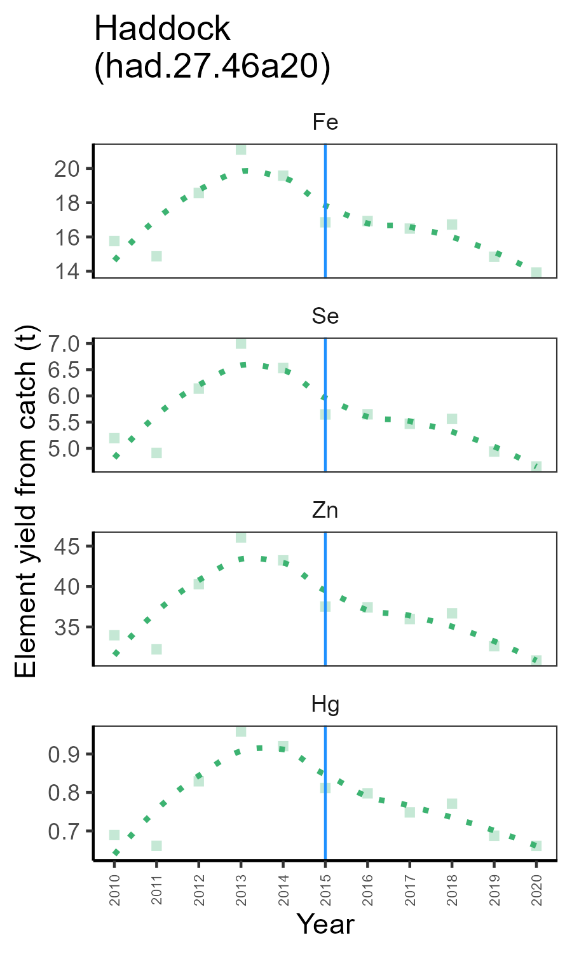


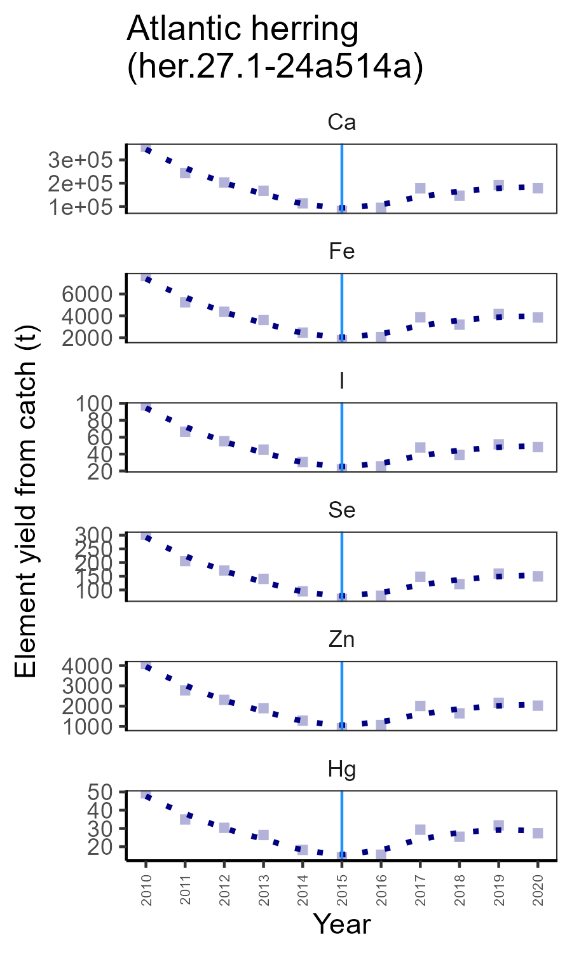

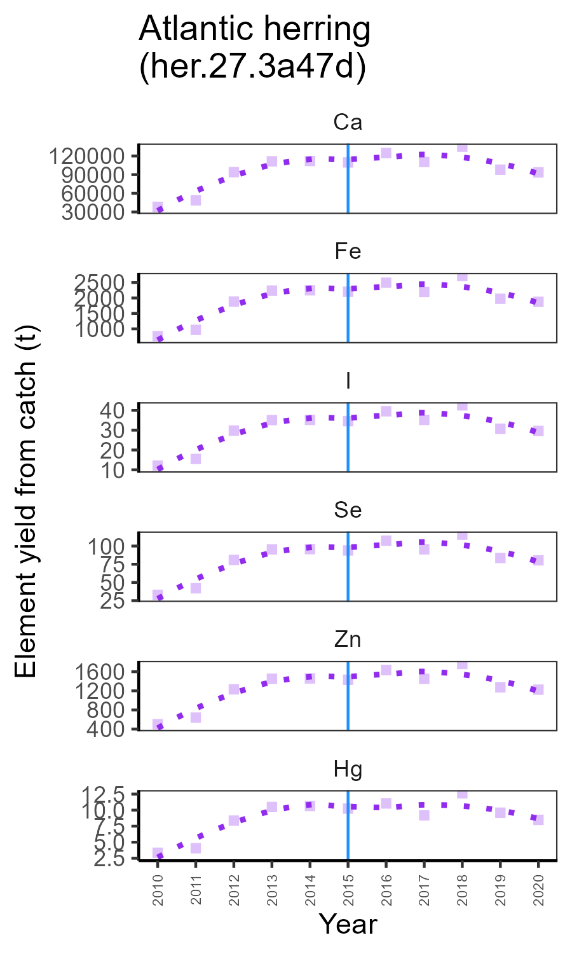


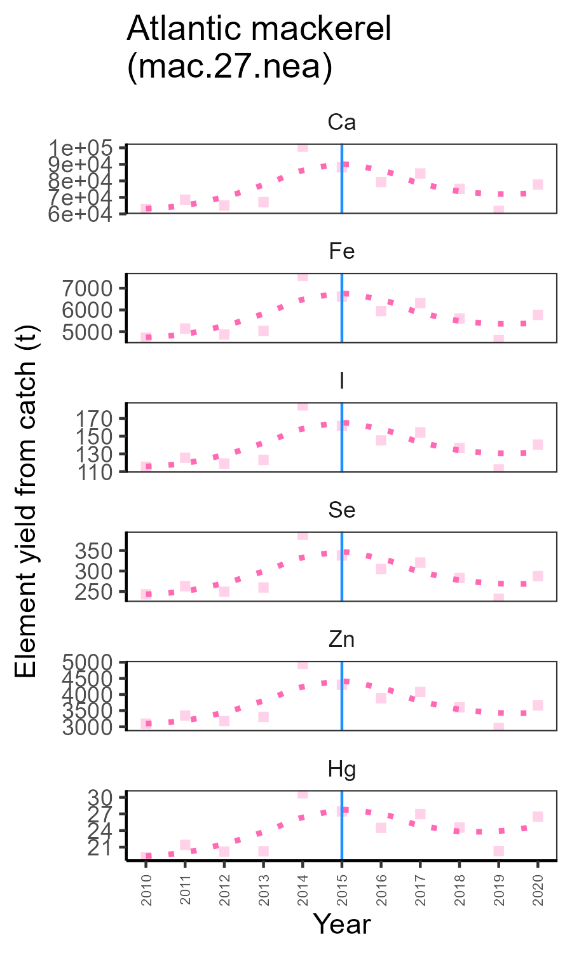


Fig. S3. Estimated annual total yields of elements for major fish stocks in the NEAO between 2010 and 2020. The trend was analysed using Loess. The blue vertical line indicates year 2015. For the stock code, cod.27.1-2: Northeast Arctic cod; cod.27.1-2.coastN: northern Norwegian coastal cod; had.27.1-2: Northeast Arctic haddock, had.27.46a20: haddock from North Sea, West of Scotland, and Skagerrak; her.27.1-24a514a: Norwegian spring-spawning herring; her.27.3a47d: autumn spawning herring from North Sea, Skagerrak, Kattegat, and eastern English Channel; and mac.27.nea: mackerel from the Northeast Atlantic and adjacent waters.


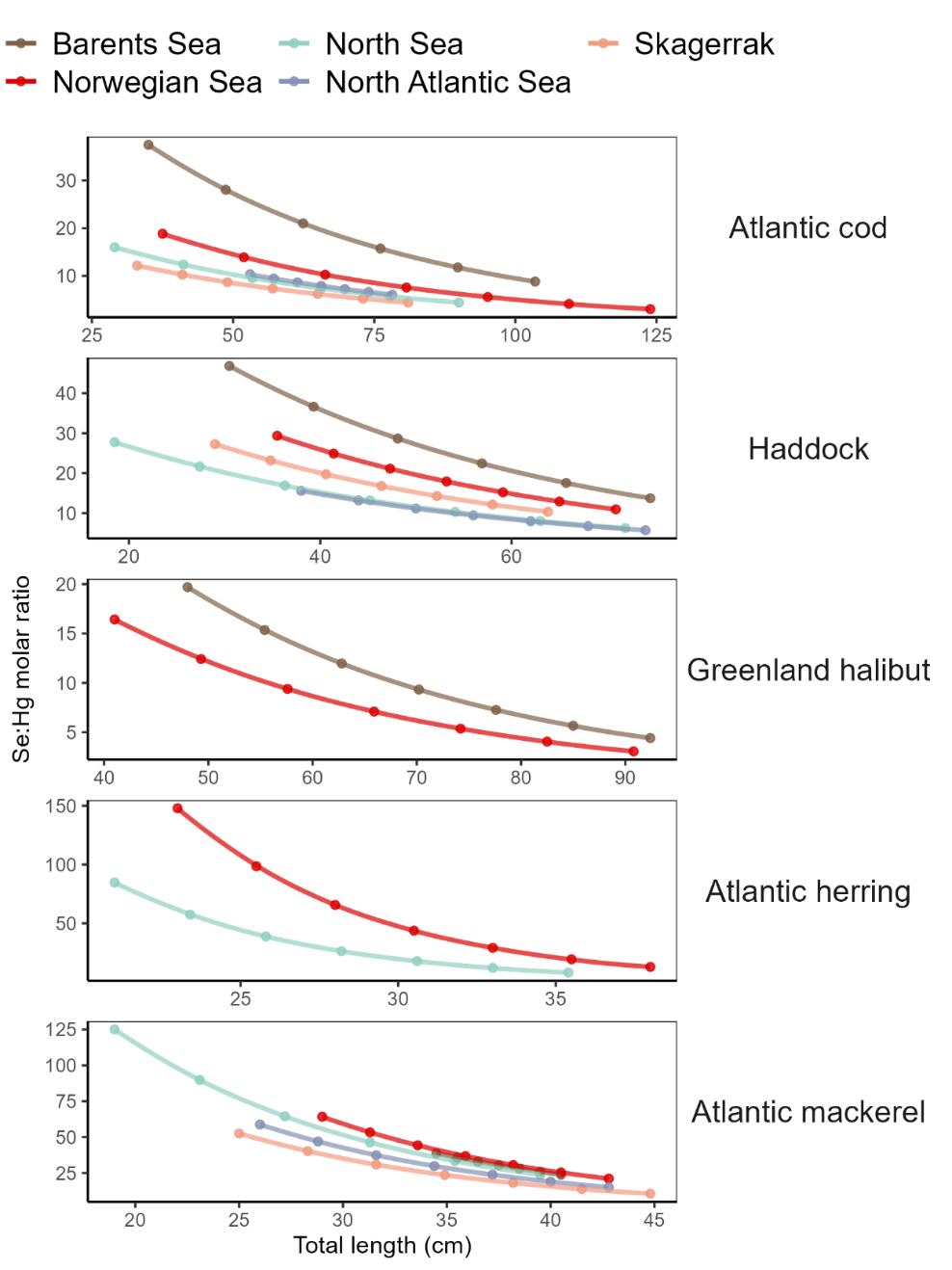


Fig. S4. Relationship between model-predicted selenium to mercury molar ratio (Se:Hg) and total length. Se:Hg molar ratio is calculated following:

$Se:Hg molar ratio={Se}_{M}\div{Hg}_{M}$ (Eq. S1)

where ***_M_*** indicates element concentration in molar, and the molar weights for Se and Hg are 78.96 and 200.59 g/mol, respectively.

**Tables:**

See separate file for Table S1-S10 (“Supplementary Materials_Tables_Zhu et al.xlsx”).

Table S1. List of abbreviations.

Table S2. Summary of (a) ranges of parameters for the predictive models (unstandardised) and (b) the output coefficients / effect sizes (mean ± 1 SD) from the predictive models (unstandardised).

Table S3. (a) Range of predictor values for model prediction of element concentrations presented in Fig. 1, (b) concentrations of nutrients and contaminants in wet weight from published sources, (c) range of predictor values for model prediction of element concentrations based on the total length categories in the extracted ICES data (Table S7), and (d) stock description of major fish stocks in the Northeast Atlantic Ocean wild catch fisheries.

Table S4. (a) Annual trade of fish mass and associated nutrient and contaminant masses (regardless of use) by species, (b) annual total nutrient and contaminant masses from all 5 fish species combined, (c) accumulative total nutrient and contaminant masses between 2010-2020, and ranked accumulative export (d) and import (e) of nutrient and contaminant masses between 2010-2020.

Table S5. Food Balance Sheet data from FAO FishStatJ and FAOSTAT (liveweight). P: production quantity, FIs: food imports, FEs: food exports, TFS: total food supply, NFUs: non-food uses, SVs: stock variations, I: import, E: export, Fee: feed, Foo: food, OUs: other uses (non-food), SV: stock variation, and Rs: residuals.

Table S6. (a) Average requirements (ARs) for nutrients and (b) daily mercury exposure per capita.

Table S7. Size structure of catch for the major fish stocks in the Northeast Atlantic Ocean.

Table S8. Mean annual percentage of total fish import for direct domestic human consumption (Γ) for two fish groups (demersal and pelagic) between 2010-2019. Lower bound (LB), middle bound (MB), and upper bound (UB) values are presented.

Table S9. Contribution of (a) traded nutrient for direct human consumption to the total domestic requirement, (b) traded mercury for direct human consumption to the total mercury exposure (Tot.exp.) and exposure from marine fish consumption (Mar.fish.exp), and (c) estimated annual per capita exposure of dioxin+dl-PCBs via traded fish for direct human consumption. Lower bound (LB), middle bound (MB), and upper bound (UB) values are presented.

Table S10. Import of other fish parts from relevant NEAO countries to Nigeria between 2011-2024.
